# Transcriptomic profile of leg muscle during early growth in chicken

**DOI:** 10.1371/journal.pone.0173824

**Published:** 2017-03-14

**Authors:** Qian Xue, Genxi Zhang, Tingting Li, Jiaojiao Ling, Xiangqian Zhang, Jinyu Wang

**Affiliations:** 1 College of Animal Science and Technology, Yangzhou University, Yangzhou, Jiangsu, China; 2 Key Laboratory for Animal Genetics, Breeding, Reproduction and Molecular Design of Jiangsu Province, Yangzhou, Jiangsu, China; Kumamoto University, JAPAN

## Abstract

The early growth pattern, especially the age of peak growth, of broilers affects the time to market and slaughter weight, which in turn affect the profitability of the poultry industry. However, the underlying mechanisms regulating chicken growth and development have rarely been studied. This study aimed to identify candidate genes involved in chicken growth and investigated the potential regulatory mechanisms of early growth in chicken. RNA sequencing was applied to compare the transcriptomes of chicken muscle tissues at three developmental stages during early growth. In total, 978 differentially expressed genes (DEGs) (fold change ≥ 2; false discovery rate < 0.05) were detected by pairwise comparison. Functional analysis showed that the DEGs are mainly involved in the processes of cell growth, muscle development, and cellular activities (such as junction, migration, assembly, differentiation, and proliferation). Many of the DEGs are well known to be related to chicken growth, such as *MYOD1*, *GH*, *IGF2BP2*, *IGFBP3*, *SMYD1*, *CEBPB*, *FGF2*, and *IGFBP5*. KEGG pathway analysis identified that the DEGs were significantly enriched in five pathways (P < 0.1) related to growth and development: extracellular matrix–receptor interaction, focal adhesion, tight junction, insulin signaling pathway, and regulation of the actin cytoskeleton. A total of 42 DEGs assigned to these pathways are potential candidate genes inducing the difference in growth among the three developmental stages, such as *MYH10*, *FGF2*, *FGF16*, *FN1*, *CFL2*, *MAPK9*, *IRS1*, *PHKA1*, *PHKB*, and *PHKG1*. Thus, our study identified a series of genes and several pathways that may participate in the regulation of early growth in chicken. These results should serve as an important resource revealing the molecular basis of chicken growth and development.

## Introduction

Chicken growth is an important economic trait for the broiler industry. A high growth rate has been one of the main objectives of broiler breeding for a long time [1]. However, the growth rate of chicken also changes as the bird develops. A growth curve is an effective way of depicting the evolution of weight with age [2]. At present, in studies on birds, three nonlinear growth models, namely, logistic, Gompertz, and von Bertalanffy, are usually used to research the growth and development patterns of birds [3–5]. The early growth pattern, especially the age of peak growth of broilers, affects the time to market and slaughter weight, which in turn affect the profitability of the poultry industry. Many studies have focused on chicken growth and some candidate genes associated with such growth, such as GH, MYOD, and IGFBP2, have been identified [6, 7]. However, few studies have investigated the mechanisms regulating growth patterns in chickens. Since such growth is controlled by multiple genes, a more systematic understanding of the genes expressed at different growth stages in chicken is needed. As a means of meeting this need, the development of RNA sequencing (RNA-Seq) technology now provides the opportunity to assess global changes in the transcriptome and to understand the regulatory pathways during the early growth of chicken [8, 9].

In this study, Jinghai Yellow chicken, a national cultivated quality broiler breed in China, was used to study the transcriptomic changes during the early growth stages. This breed was derived from native chickens in Nantong city Jiangsu province in China according to traditional genetic breeding theory after the selection of seven generations. Compared to the fast-growing breed introduced from abroad and some other national cultivated quality broiler breed, Jinghai Yellow chicken is a slow-growing breed characterized by small body size, the adult weight of which was only about 1200g. Another character of this breed is early maturity, because its age at the first egg is very young, which is about 130 days old. And since the native chickens’ adaptability to the severe environment outdoors and poor quality feeds over time, a strong stress resistance has been formed in Jinghai Yellow chicken. After seven generations of breeding, the variable coefficient of the weight in the population was becoming lower and lower [10]. A previous study showed that the accumulated weight curve of Jinghai Yellow chicken is a typical S-curve, and the growth pattern involves slow growth before the age of 4 weeks, then increasingly rapid growth until a peak at about 12 weeks, followed by a decline in growth until a fully grown state is reached at 23 weeks. Upon fitting a growth curve for Jinghai Yellow chicken, the inflexion point for growth was shown to occur at 11.42 weeks of age [11]. This breed has been used for studies on chicken growth and carcass traits [12, 13]. A genome-wide association study on this breed showed that a total of 18 single-nucleotide polymorphisms (SNPs) were significantly (P < 1.80E^−6^) associated with growth traits [14]. Therefore, in the present study, Jinghai Yellow chickens at 4 weeks of age (slow-growing stage), 12 weeks of age (fast-growing stage), and 16 weeks of age (growth retardation/on-sale stage) were selected to study the changes in gene expression at the genome-wide level during the early growth stages and then to identify candidate genes involved in chicken growth.

Most researches on chicken growth and development in the past decades mainly focused on the breast muscle [15, 16], but rare information on leg muscle transcriptome exists. And the broiler industry has been very successful in selecting broilers with increased breast yields. However, the regulatory mechanism about the growth and development of chicken leg muscle are neglected. Therefore, the present study was carried out to study the transcriptomic changes in leg muscle during early growth stages. RNA-Seq technology and bioinformatic tools were used to investigate the major differentially expressed genes (DEGs) and pathways. In addition, qPCR experiments were performed to validate the RNA-Seq results. The results of this study are useful for understanding the mechanisms regulating the development of leg muscle and the pattern of chicken growth. And the findings should provide a basis for increasing chicken leg muscle yields for broiler industry.

## Materials and methods

### Ethics statement

All animal experiments were performed in accordance with the protocol of the Animal Use Committee of the Chinese Ministry of Agriculture, and were approved by the Animal Care Committee of the Department of Animal Science and Technology, Yangzhou University. All efforts were made to minimize animal suffering.

### Animals and tissues

The chickens in this study were obtained from Jiangsu Jinghai Poultry Industry Group Co., Ltd. (Nantong City, Jiangsu Province, China). Birds were fed *ad libitum* with formula feed for Jinghai Yellow chickens, and were given free access to water. The body weight (BW) was measured every week from 0 to 25 weeks of age to analyze the pattern of growth of Jinghai Yellow chickens. Using the average values of BW at 4, 12, and 16 weeks of age (188.73±6.25, 958.0±19.84, and 1257.33±24.4 g, respectively), three female chickens with BW values around these averages were selected at each of these stages. Therefore, nine female chickens from the three different growth stages were used for the RNA-Seq. The selected chickens were killed at 4, 12, and 16 weeks of age by stunning followed by exsanguination. Leg muscles were then collected immediately, snap-frozen in liquid nitrogen, and stored at −80°C until RNA extraction.

### Statistical analysis

The SPSS 17.0 software package (SPSS Inc., Chicago, IL, USA) was used to analyze the BW data. The three nonlinear growth models of logistic, Gompertz, and von Bertalanffy were used to fit growth curves of Jinghai Yellow chicken [3, 17]. The equations of these three nonlinear models were as follows:

Logistic: $y_{it}=A_{i}{\left[1+B_{i}{}^{\left(-K_{i}\times t\right)}\right]}^{{}_{-1}}+\mu_{i}+\varepsilon_{it}$
Von Bertalanffy: $y_{it}=A_{i}{\left[1-B_{i}{}^{\left(-K_{i}\times t\right)}\right]}^{3}+\mu_{i}+\varepsilon_{it}$
Gompertz: $y_{it}=A_{i}\text{exp}\left[-B_{i}{}^{\left(-K_{i}\times t\right)}\right]+\mu_{i}+\varepsilon_{it},$

in which *y*_*it*_ is the observed BW of the *i*th animal at age *t*, *A*_*i*_ is the asymptotic weight for the *i*th animal, *B*_*i*_ is the integration constant, *K*_*i*_ is the maturing rate of the *i*th animal, *t* is the age in days, *μ*_*i*_ is the random effect for animal *i*, and *ε*_*it*_ is the fitting error [18]. GraphPad Prism 5.0 software (GraphPad Software Inc., San Diego, CA, USA) was used to draw the growth curve.

### Total RNA extraction and RNA-Seq library construction

Three individuals from each growth stage were used for the RNA-Seq analysis. Total RNA from the leg muscles was isolated using the TRIzol total RNA extraction kit (Invitrogen, Carlsbad, CA, USA), in accordance with the manufacturer’s instructions. The concentration and purity of RNA were evaluated using a Nanodrop 2000c Spectrophotometer (Thermo Scientific) and the integrity was determined using an Agilent 2100 Bioanalyzer (Agilent Technologies, Santa Clara, CA, USA). mRNA libraries were constructed using the TruSeq RNA Sample Preparation Kit (Illumina, Inc., San Diego, CA, USA), in accordance with the TruSeq protocol and then sequenced on a single lane using an Illumina X10 (Illumina, Inc.) platform.

### Bioinformatic analysis of RNA-Seq data

Using FASTQC (http://www.bioinformatics.babraham.ac.uk/projects/fastqc/), the quality of the raw data was assessed. Low-quality reads (reads containing adaptors, unknown bases, and low-quality bases) were removed using NGSQC Toolkit v2.3.3 [19]. The clean reads obtained after data filtering were mapped to the chicken reference genome (ftp://ftp.ncbi.nlm.nih.gov/genomes/all/GCF_000002315.4_Gallus_gallus-5.0/GCF_000002315.4_Gallus_gallus-5.0_genomic.fna.gz) using TopHat software. The mapped reads were used for further transcript annotation and for calculating the expression level using the FPKM (fragments per kilobase per million reads) method. The DESeq package (http://bioconductor.org/packages/release/bioc/html/DESeq.html) was used to calculate the difference in gene expression [20]. Genes with false discovery rate (FDR) < 0.05 and fold change ≥ 2 were considered to be DEGs between two groups. All of these DEGs were then subjected to Gene Ontology (GO) annotation and enrichment analysis (ftp://ftp.ncbi.nlm.nih.gov/genomes/all/GCF_000002315.4_Gallus_gallus-5.0/GCF_000002315.4_Gallus_gallus-5.0_genomic.gff) by the classic algorithm and Fisher’s exact test. For further characterization of the metabolic pathways of DEGs, the Kyoto Encyclopedia of Genes and Genomes (KEGG) database was used to analyze the pathways [21].

### Validation of RNA-Seq results

The expression of 9 genes was quantified by qPCR to validate the RNA-Seq data. Primer pairs used for quantification were designed using Primer Premier 5.0 software (PREMIER Biosoft, California, United States), covering different exons in order to assure the amplification of the cDNA(S1 Table). The validation was performed on the same three samples at each growth stage used for the RNA-Seq analysis, as three biological replicates. Total RNA was reverse-transcribed into cDNA using the PrimeScript^™^ RT Master Mix kit (TaKaRa Biotechnology Co Ltd, Dalian, China). qPCR was conducted on an Applied Biosystems 7500 real-time PCR system (Applied Biosystems) in a total volume of 20 μL including 10 μL of SYBR^®^ Premix Ex Taq^™^ (2×), 0.4 μL of ROX Reference Dye II (TaKaRa Biotechnology Co Ltd, Dalian, China), 0.4 μL of each primer (10 μM), 6.8 μL of RNase-free water and 2 μL of cDNA.

The PCR efficiency of each primer pair was calculated by the standard curve method using five points of cDNA serial dilutions. The cDNA template used for this purpose came from a pool of 9 cDNAs from the 9 samples used for RNA-Seq. PCR efficiency (E) was calculated as follows: E = (10^(1/−*S*)^ − 1) × 100, where S is the slope from the standard curve [22]. The transcription stability of ACTB, TBP and YWHAZ genes was calculated in previous studies, and these three genes were recommended as the most suitable reference genes for the quantitative PCR analysis of growth [23–25]. Therefore, in the present study, ACTB, TBP and YWHAZ genes were selected as internal reference genes for normalization of the expression data. The relative expression levels of the target genes were calculated with the normalization factors based on the geometric means of these three reference genes quantities. The 2^−ΔΔCt^ method was used to transform the data from the relative quantification [26]. The PCR reaction program was as follows: initial denaturation at 95°C for 30 s, and 40 cycles of denaturation at 95°C for 5 s and annealing at 60°C for 34 s.

## Results

### Early growth and development pattern in Jinghai Yellow chicken

**Fig 1:**
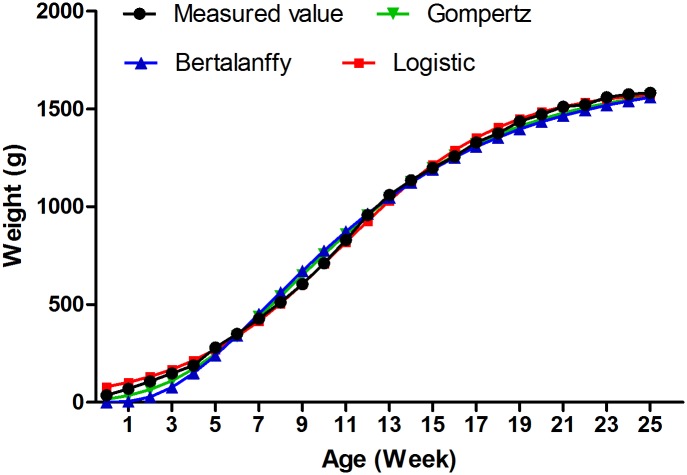
The actual growth curve and the matching curve of Jinghai Yellow chicken.

The results showed that the growth curve of Jinghai Yellow chicken resembled an S-curve. Specifically, it was shown to grow very slowly from 0 to 4 weeks of age. Its peak growth occurred between 9 and 13 weeks of age, and then its rate of growth declined until a fully grown state was reached at 23 weeks of age (S2 Table, Fig 1). The parameter estimates of the three growth models are presented in Table 1. All of the three models well described the growth patterns of Jinghai Yellow chicken (R^2^ > 0.99). The value of R^2^ in the logistic model reached 0.999. In addition, in the growth curve fitted by the logistic model, the age and BW at the inflexion point of growth were respectively 11.48 weeks and 796.68 g. The fitting results closely matched the measured values.

**Table 1:** Results of the estimates for the three nonlinear models.

Model	A	B	K	R^2^*	WGI^a^	BGI^b^
Gompertz	1730.52	4.26	0.16	0.999	9.06	636.62
Logistic	1593.36	19.81	0.26	0.999	11.48	796.68
Von Bertalanffy	1837.42	0.88	0.12	0.997	8.13	544.42

*R^2^ represents the goodness of fit.

^a^WGI indicates the weeks of age at the inflexion point of growth.

^b^BGI indicates the body weight at the inflexion point of growth.

### Blast analysis of reads from RNA-Seq

We established nine cDNA libraries, three from each growth stage (including 4, 12, and 16 weeks of age), to identify DEGs related to growth and development by RNA-Seq. Detailed results of the sequencing and Blast analysis are shown in Table 2. More than 60 million raw reads were obtained for each individual sample. After filtering the low-quality reads, the average numbers of clean reads were 58,362,898 (93.9%), 61,924,767 (94.4%), and 59,191,757 (94.6%) for the three groups, respectively. All sequencing data have been submitted to NCBI Sequence Read Archive (SRA) with the accession number SRR4296893. Upon mapping to the chicken reference genome, averages of 41,704,217, 44,786,644, and 45,134,724 total mapped reads were obtained for the three groups, with mapping ratios of 71.5%, 72.3%, and 76.3%, respectively. The distribution of the mapped reads in different regions of the chicken reference genome showed that most of the reads had been mapped to the coding sequence of exons and introns (S1 Fig).

**Table 2:** Data summary from RNA-seq.

Sample^1^	Raw reads	Clean reads	Clean bases	Valid ratio (%)	Q30(%)	GC content (%)	Total mapped reads	Mapping ratio (%)^2^
M4W-1	63390474	59877776	8976603064	94.40	92.44	50.50	42743796	71.39
M4W-2	62481624	59772130	8961396183	95.61	93.81	50.50	42391042	70.92
M4W-3	60332474	55438788	8310479464	91.82	89.62	49.50	39977813	72.11
M12W-1	64907194	60904330	9130228864	93.77	91.73	50.50	43150444	70.85
M12W-2	67880554	64278528	9636498748	94.64	92.71	50.50	46253368	71.96
M12W-3	63800848	60591442	9083866415	94.91	92.37	49.50	44956120	74.20
M16W-1	60207798	57616766	8638195009	95.64	93.86	49.50	43937315	76.26
M16W-2	63781302	60234872	9030243563	94.38	93.04	50.50	46392239	77.02
M16W-3	63639886	59723634	8953287453	93.79	91.74	50.00	45074618	75.47

^1^M4W, M12W, and M16W indicate muscle samples at 4, 12, and 16 weeks of age, respectively.

^2^Mapping ratio indicates the level of total mapped reads as a proportion of clean reads.

**Fig 2:**
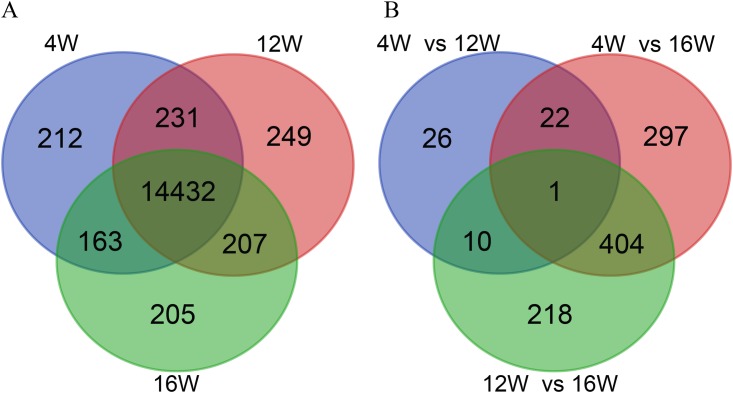
Numbers of expressed genes and differentially expressed genes: Results of RNA-Seq. A: Expressed genes among the three groups. B: Differentially expressed genes among three comparisons, namely, 4W vs. 12W, 4W vs. 16W, and 12W vs. 16W. 4W, 12W, and 16W indicate the chicken groups at 4, 12, and 16 weeks of age, respectively.

**Fig 3:**
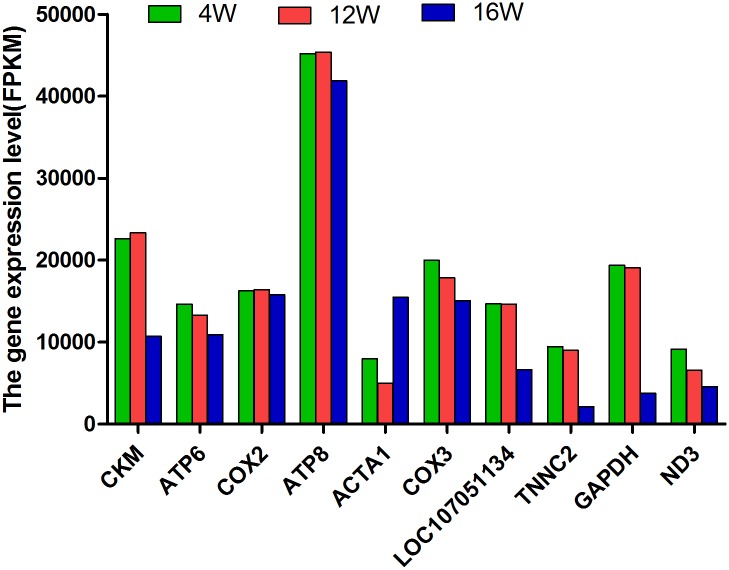
Top ten most abundantly expressed genes in chicken leg muscles at 4, 12, and 16 weeks of age.

In total, 15,699 genes were detected in all of the samples, including 15,038 in the samples of chicken muscle at the age of 4 weeks, 15,119 in the samples at the age of 12 weeks, and 15,007 in the samples at the age of 16 weeks. Among these genes, 14,432 were commonly expressed in all three groups, while 212, 249, and 205 genes were found exclusively at 4, 12, and 16 weeks of age, respectively (Fig 2A). The top ten most abundantly expressed genes in the three groups (FPKM from 10,507 to 51,939 reads) ranked by absolute abundance were CKM (muscle-specific creatine kinase), ATP6 (ATP synthase protein 6), COX2 (cyclooxgenase-2), ATP8 (ATP synthase protein 8), ACTA1 (actin alpha 1), COX3 (cyclooxgenase-3), LOC107051134 (creatine kinase M-type-like), TNNC2 (troponin C type 2), GAPDH (glyceraldehyde-3-phosphate dehydrogenase), and ND3 (NADH dehydrogenase subunit 3). The expression levels of TNNC2 and GAPDH were much lower at 16 weeks of age than at 4 and 12 weeks of age (Fig 3).

### Differentially expressed genes among the three groups

**Fig 4:**
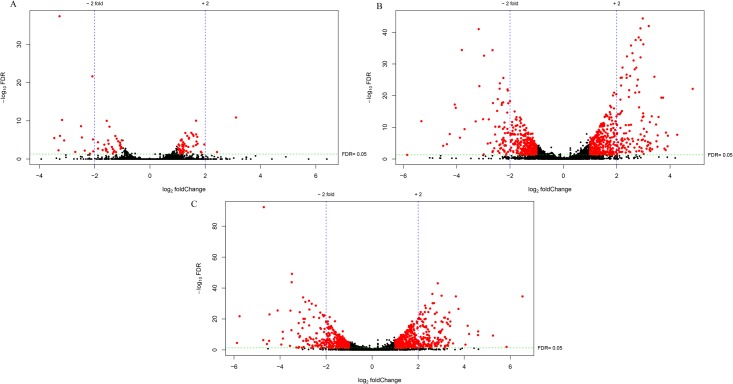
Differentially expressed genes identified in the comparisons of 4W vs. 12W (A), 4W vs. 16W (B), and 12W vs. 16W (C).

A comparison of gene expression among the three groups showed that there were 59 DEGs (fold change ≥ 2; FDR < 0.05) between 4 and 12 weeks of age, 724 DEGs between 4 and 16 weeks of age, and 633 DEGs between 12 and 16 weeks of age (S3 Table, Fig 2B). The DEGs from each comparison are also shown in red in a volcano plot (Fig 4). Differential expression of FGF16 (fibroblast growth factor 16) was found in all comparisons (Fig 2B), which indicated that FGF16 expression was changing throughout the entire period of early growth. Among all of the DEGs, 5, 90, and 77 genes were uniquely expressed in one of the two groups in the comparisons of 4W vs. 12W, 4W vs. 16W, and 12W vs. 16W, respectively (S4 Table), which included some genes that are important for growth and development, such as GH, IGF2BP3, DMP1, HoxC8, MEPE, and SSTR2. DEG directionality analysis showed that there were 32, 408, and 360 upregulated genes, and 27, 316, and 273 downregulated ones, respectively, in the comparisons of 4W vs. 12W, 4W vs. 16W, and 12W vs. 16W (S2 Fig). The number of upregulated genes was higher than the number of downregulated genes in all of the comparisons.

### GO enrichment analysis for DEGs

DEGs were then used for GO analysis to uncover their functional enrichment in each comparison. These DEGs were categorized into three main GO categories of biological process, cellular component, and molecular function. In total, there were 20, 163, and 159 significantly enriched GO terms (P < 0.05) identified in 4W vs. 12W, 4W vs. 16W, and 12W vs. 16W, respectively (S5 Table). Many of the significantly enriched biological process terms in the comparisons of 4W vs. 16W and 12W vs. 16W are associated with cell growth and development, such as regulation of cell growth, multicellular organism growth, muscle organ development, actin filament organization, and actin cytoskeleton organization. A total of 39 DEGs were found for these GO terms, including genes well known to affect chicken growth, such as IGFBP3, MYOD1, IGFBP5, and IGFBP7 (Table 3). In this study, the genes with differential expression among the different stages of development were also found to be significantly enriched for terms related to the processes of cell activities, including cell junction, migration, assembly, differentiation, and proliferation. Thirty-eight DEGs were associated with these terms, with some of these genes having been reported to be associated with growth, such as SMYD1, IGFBP3, MYOD1, CEBPB, FGF2, and IGFBP5 (Table 4). In particular, among the GO terms mentioned above, several terms associated with muscle development were included, such as skeletal muscle fiber development, muscle organ development, and skeletal muscle cell differentiation. In addition, 10 genes were included in these processes (Table 5). These genes might be crucial for muscle development and chicken growth.

**Table 3:** Significantly enriched biological process terms involved in cell growth and development.

Comparison	Term ID	Term	Genes	*P*-value
4W vs. 16W	GO:0001558	regulation of cell growth	IGFBP3; KAZALD1; SOCS2	0.04903
	GO:0007528	Neuromuscular-junction development	PDZRN3; MUSK; CHRNA1; COL4A5; UNC13A	0.00036
	GO:0048741	skeletal muscle fiber development	KLHL40; GPX1; PPP3CA; MYOD1	0.00131
	GO:0007517	muscle organ development	CRYAB; MYOD1; TCF15	0.00615
	GO:0007010	cytoskeleton organization	RHOU; ABLIM1; ABLIM2; LIMD1; ABLIM3; MICAL2; MICAL3;	0.00575
	GO:0030036	actin cytoskeleton organization	RHOU; WDR1; MYH10; ABLIM2; ABLIM3; PFN2; DNAJB6	0.03871
12W vs. 16W	GO:0007528	neuromuscular-junction development	PDZRN3; MUSK; CHRNA1; COL4A1; UNC13A	0.00019
	GO:0001558	regulation of cell growth	IGFBP3; IGFBP5; IGFBP7; ESM1	0.00719
	GO:0035264	multicellular organism growth	TAL2; EN1; GPD2; SLC12A5; SLC1A2; XPA	0.01639
	GO:0007015	actin filament organization	SORBS1; CFL2; MYO1B; FHOD3	0.01996
	GO:0030036	actin cytoskeleton organization	RHOU; WDR1; MYH10; ABLIM2; ABLIM3; PFN2; DNAJB6	0.02101

**Table 4:** Significantly enriched terms related to the processes of cellular activities.

Comparison	Term ID	Term	Genes	*P*-value
4W vs. 12W	GO:0001649	osteoblast differentiation	MYOC; COL6A1; SNAI2	0.00029
	GO:0030335	positive regulation of cell migration	MYOC; LRRC15; SNAI2	0.00075
4W vs. 16W	GO:0055003	cardiac myofibril assembly	MYH10; TCAP; CSRP3	0.00032
	GO:0045662	negative regulation of myoblast differentiation	CSRP3; ANKRD2; TBX3; IL18	0.00131
	GO:0014902	myotube differentiation	BARX2; GPX1; MYOD1	0.00135
	GO:0045663	positive regulation of myoblast differentiation	IGFBP3; CSRP3; SMYD1	0.00135
	GO:0048662	negative regulation of smooth muscle cell proliferation	TNFAIP3; IGFBP3; PPARG	0.00479
	GO:0035914	skeletal muscle cell differentiation	BTG2; MYOD1; ANKRD2; SMYD1; MYLK2	0.01168
	GO:0051496	positive regulation of stress fiber assembly	MYOC; SERPINF2; PFN2	0.04388
12Wvs. 16W	GO:0035914	skeletal muscle cell differentiation	BTG2; MYOD1; ANKRD2; SMYD1	0.02715
	GO:0030239	myofibril assembly	MYH10; TMOD1; CAPN3	1.05E^−10^
	GO:0045663	positive regulation of myoblast differentiation	IGFBP3; CSRP3; SMYD1; PLCB1	5.30E^−05^
	GO:0030837	negative regulation of actin filament polymerization	PFN2; TWF1; FHOD3	0.00020
	GO:0055003	cardiac myofibril assembly	MYH10; CSRP3; FHOD3	0.00020
	GO:0060644	mammary gland epithelial cell differentiation	FGF2; ZNF703; CEBPB	0.00057
	GO:0048662	negative regulation of smooth muscle cell proliferation	TNFAIP3; GFBP3; IGFBP5	0.00313
	GO:0045662	negative regulation of myoblast differentiation	CSRP3; ANKRD2; IL18	0.00633
	GO:0045668	negative regulation of osteoblast differentiation	LIMD1; IGFBP5; HAND2	0.03025

**Table 5:** Differentially expressed genes involved in the processes of muscle development in the three comparisons.

Gene	Description	Gene	Description
MYOD1	myogenic differentiation 1	PPP3CA	protein phosphatase 3, catalytic subunit, alpha isozyme
MYLK2	myosin light chain kinase 2	GPX1	glutathione peroxidase 1
SMYD1	SET and MYND domain containing 1	TCF15	transcription factor 15 (basic helix-loop-helix)
BTG2	BTG family, member 2	KLHL40	kelch-like family member 40
ANKRD2	ankyrin repeat domain 2 (stretch responsive muscle)	CRYAB	crystallin, alpha B

### KEGG pathway analysis for DEGs

KEGG enrichment analysis of DEGs showed that 24, 221, and 191 genes were annotated in the KEGG database for the comparisons of 4W vs. 12W, 4W vs. 16W, and 12W vs. 16W, respectively. In addition, there were 3, 19, and 18 significantly enriched KEGG pathways (*P* < 0.1), respectively, in these three comparisons (S6 Table). Many of these pathways are related to the metabolism of amino acids, carbohydrates, and lipids, such as glycine, serine, and threonine metabolism; pyruvate metabolism; the pentose phosphate pathway; the PPAR signaling pathway; and glycolysis/gluconeogenesis, among others. Five significantly enriched pathways related to growth and development were identified, namely, extracellular matrix (ECM)–receptor interaction, focal adhesion, tight junction, insulin signaling pathway, and regulation of the actin cytoskeleton (Table 6). There were 42 DEGs associated with these five pathways.

**Table 6:** Pathways related to growth and development.

Comparisons	Pathways	*P* value	Genes
4W vs. 12W	ECM–receptor interaction	0.0561	COL6A3, COL6A1, FN1
	Focal adhesion	0.0710	COL6A3, COL6A1, PIK3R3, FN1
4W vs. 16W	Insulin signaling pathway	0.0124	SOCS2, PHKG1, PHKB, PHKA1, FBP2, PPP1R3A, IRS1, PRKAR2B, EIF4EBP1, PPP1R3C, CRKL, SORBS1, MAPK9
	Tight junction	0.0127	PRKCQ, MYH1E, EPB41L1, MYH1F, VAPA, MYL10, MYH1D, MYH1C, MYH7B, MYH1B, MYH10, MYH1A
12W vs. 16W	Regulation of actin cytoskeleton	0.0446	ARHGEF4, MYLK3, FGF16, MYLK4, BDKRB1, FGF13, MYL10, MYL12A, FGF12, PFN2, CFL2, ITGB6, FGF2, FN1
	Tight junction	0.0314	MYH1E, EPB41L1, PPP2CA, MYL10, MYL12A, MYH1C, MYH7B, MYH1B, MYH10, MYH1A

**Fig 5:**
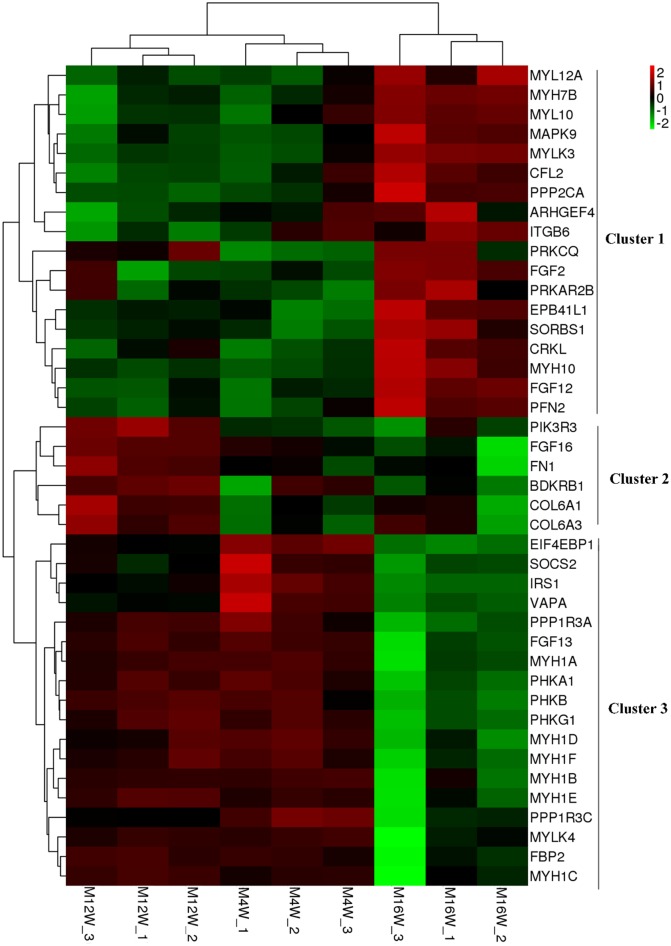
Heatmap of differently expressed genes in the five pathways related to chicken growth. Rows indicate genes with significant differences in expression among the three stages; columns represent individual samples from three stages (M4W, M12W, and M16W indicate muscle samples at 4, 12, and 16 weeks of age, respectively.).

Hierarchical clustering analysis was applied to compare the expression patterns of these 42 DEGs at the early growth stages (Fig 5). In the heatmap, different colors represent different expression levels. Red represents the highest expression level and green represents the lowest. In this map, the expression patterns of these genes could be roughly divided into three clusters. Cluster 1 was enriched in genes that were significantly upregulated at 16 weeks of age compared with those at 4 and 12 weeks of age. This cluster included 18 DEGs, such as FGF2, MYH10, FGH12, CFL2, and ITGB6. Cluster 2 consisted of 6 DEGs, namely, PIK3R3, FGF16, FN1, BDKRB1, COL6A1, and COL6A3. The expression levels of these genes were the highest at 12 weeks of age. The expression pattern of cluster 3 was completely the opposite of that of the first cluster, being enriched in genes downregulated at 16 weeks of age compared with those at 4 and 12 weeks of age. There were 18 DEGs in this cluster, including EIF4EBP1, SOCS2, IRS1, VAPA, MYH1s, PHKs, and FGF13. In addition, the results of sample clustering showed that three samples from the same stage were classified into the same cluster, and samples from different stages were differentiated, which confirmed the reliability of the sampling in this study.

### qPCR validation of DEGs obtained from RNA-Seq

**Fig 6:**
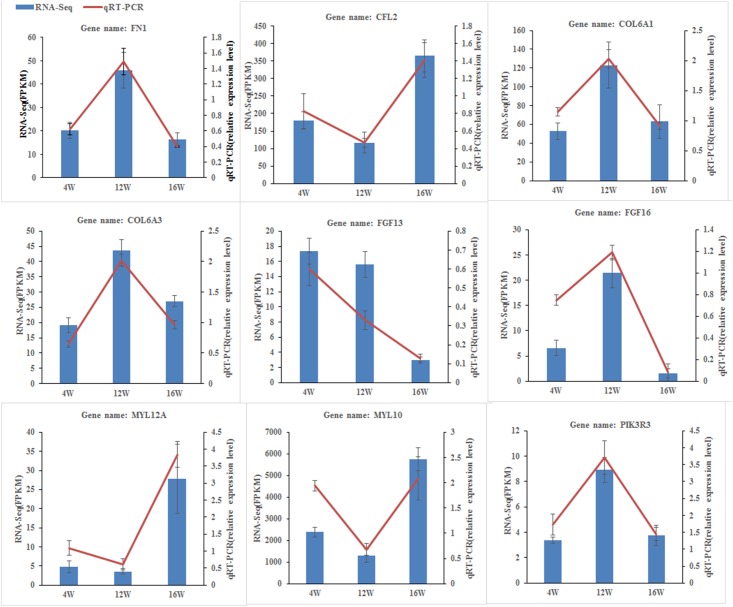
Expression level of nine DEGs detected by RNA-Seq and validated by qPCR. Results from RNA-Seq are shown by bar graphs and values are shown on the right y-axis as FPKM. Results from qPCR are shown by line graphs and values are shown on the left y-axis as relative expression level. Data are presented as mean±SE.

Nine DEGs from the five significantly enriched pathways related to growth (Table 6) were randomly selected to validate the results of RNA-Seq. qPCR was carried out on the same RNA samples as used for RNA-Seq. The PCR efficiency of each primer pair was estimated by standard curve calculation. And the results showed that the efficiency values of all the primers in this study were around 100%, and all the coefficients of determination (R^2^) were above 0.98 (S3 Fig), which indicated that the qPCR assays have been optimized and the results of quantification were accurate and reliable. After qPCR analysis of the nine target genes, it was found that the qPCR results were consistent with the RNA-Seq results regarding the direction of changes in the expression level of DEGs (Fig 6), which confirmed the validity of the data from RNA-Seq.

## Discussion

In the present study, the early growth pattern of Jinghai Yellow chicken was analyzed. The inflexion point of growth was determined to occur at 11.48 weeks of age. According to the growth curves, the early growth of chicken could be divided into three developmental stages: a slowly growing stage before 4 weeks of age, a rapidly growing stage between 9 and 13 weeks of age, and a growth retardation (on-sale) stage around 16 weeks of age. These results are consistent with a previous report about the pattern of growth in Jinghai Yellow chicken [11].

To identify genes causing the differences in growth rates among the three developmental stages, transcriptomes of the leg muscles were compared among these stages. Of the top ten most abundantly expressed genes (ACTA1, CKM, ATP6, ATP8, COX2, COX3, LOC107051134, TNNC2, GAPDH, and ND3) detected in the samples, most are the genes encoding enzymes (except ACTA1 and TNNC2) involved in the processes of cell energy metabolism and synthesis, glycolysis, respiration and oxidization [27–30]. ACTA1 encodes skeletal muscle alpha-actin and TNNC2 is a constituent of the troponin complex located on the thin filament, which were reported to be associated with skeletal muscle contraction [31, 32]. It is well known that these processes involved by the top ten genes are essential for maintaining the life activities of animals. Therefore, these genes might play important roles during chicken growth and development. Interestingly, GAPDH gene, one of the most commonly used housekeeping gene [33, 34], was identified as a DEG in this study because of its significantly lower expression level at 16 weeks of age than at 4 and 12 weeks of age. And in some other previous studies [35, 36], it was shown that the expression of GAPDH were not always stable in different tissues or under different experimental conditions. Therefore, the result in this study led us to suggest that GAPDH might not be used as the housekeeping gene for the growth of chicken leg muscle.

Differential expression of genes related to growth is considered to be the primary reason for genetic variation in chicken growth. In this study, the number of DEGs identified in the comparison of 4W vs. 12W was remarkably less than detected in the comparisons of 4W vs. 16W and 12W vs. 16W(Fig 4), suggesting a shift in regulatory mechanisms influencing growth between these two stages. Of all the DEGs involved in growth and development identified in this study, some were previously reported to be closely related to chicken growth, including several transcription factors, such as MYOD1, FBXO32, CEBPB, and FOXO3, and a series of genes involved in the somatotropic axis, such as GH, IGF2BP1, IGF2BP2, IGF2BP3, IGFBP3, IGFBP5, and IGFBP7 [37–39]. MYOD1, FBXO32, CEBPB, and FOXO3 are four crucial transcription factors that might be responsible for the differential expression of other growth-related genes between different growth stages. CEBPB is an activator of adipogenesis as well as an inhibitor of myogenesis [40, 41]. Previous researches showed that it regulates multiple genes in response to GH [42]. In this study, the expression of CEBPB was upregulated at 16 weeks of age compared with its expression level at 12 weeks of age, but there were no differences in the other two comparisons, suggesting that it may play an important role in growth at 16 weeks of age. A recent study demonstrated that compared to the fast-growing WRR chickens, the higher expression of CEBPB in slow-growing XH chickens might contributed to their lower growth performance, which was in agreement with the results in this study [15]. Therefore, it is possible that the higher expression of CEBPB at 16 weeks of age contributes to the lower growth rate at this stage. As another example of transcription factor identified as being differentially expressed in this study, FBXO32 is a muscle-specific gene that was shown to be associated with muscle atrophy [43]. A previous study showed that, as the muscle degraded after feed deprivation, FBXO32 expression increased [44]. FOXO3 performs a variety of cellular functions, including involvement in cell growth and differentiation, cell cycle control, energy metabolism, DNA damage repair, response to oxidative stress, and apoptosis [45–49]. It has been identified as a major activator of FBXO32 expression [50], which may explain our results that both were downregulated at 16W compared with their levels at 12W. It has been reported that, with FBXO32 downregulation, growth-related genes including PDK4, IGF2R, and IGFBP3 were significantly downregulated. In this study, the downregulation of IGFBP3, IGFBP5, IGFBP7, and IGF2BP1 at 16 weeks of age was inferred to be associated with the downregulation of FBXO32. Furthermore, studies have shown that these regulatory transcription factors can interact with each other in regulating chicken growth [15, 51].

In this study, we identified several members of IGFBP (insulin-like growth factor-binding protein) gene family, such as IGFBP3, IGFBP5 and IGFBP7. IGFBP, a family of six or more related proteins that bind IGF with high affinity, was reported to regulate the activity of IGF and influence cell growth [39, 52]. Studies have shown that IGFBPs could sequester IGF to decrease protein synthesis and inhibit muscle cell differentiation [53]. However, the expression of IGFBP3, IGFBP5, and IGFBP7 at 12 weeks of age was upregulated compared with that at 16 weeks of age, and was not significantly different from the expression at 4 weeks of age, which were the unexpected findings in this study. But other studies showed that IGFBPs exerted a variety of biological actions not involving IGFs [54, 55, 56]. For instance, IGFBP-5 was suggested to in part stimulate bone cell proliferation by an IGF-independent mechanism involving IGFBP-5-specific cell surface binding sites [54]. So it is inferred in the present study that these genes could be functioning through an alternate pathway, beyond their role in inhibiting protein synthesis through binding to IGF and blocking receptor binding to IGFRs.

Postnatal growth of skeletal muscle is mainly due to myofiber hypertrophy accompanied by the proliferation of satellite cells, which make new myonuclei incorporated into existing myofibers, resulting in an increase in DNA content and providing the machinery for protein deposition [57]. GO analysis showed that the DEGs are mainly involved in the processes of cell growth, muscle development, and cellular activities (such as junction, migration, assembly, differentiation, and proliferation), as well as muscle contraction and glycogen metabolic and biosynthetic processes. In this study, the comparison of 4W vs. 12W resulted in identification of the lowest number of DEGs, which were in turn associated with the fewest GO biological process terms; however, the terms of cell differentiation, cell migration, and development process were present in all of the comparisons. Chicken growth is a complex process influenced by multiple genes and controlled by multiple pathways. In our KEGG analysis, five pathways related to growth were identified, namely, ECM–receptor interaction, focal adhesion, tight junction, insulin signaling pathway, and regulation of the actin cytoskeleton, of which the insulin signaling pathway was the most significantly enriched.

The insulin-signaling pathway was demonstrated to be involved in translation initiation and the efficiency of the translation process directly affects the rate of protein synthesis. Insulin, as a component of this pathway, plays a key role in stimulating glucose transport [58, 59]. Recently, studies have shown that the ontogenetic changes in the expression of genes in this pathway in skeletal muscle contribute to the developmental decline in protein synthesis [60]. Thus, this pathway has been considered to be involved in growth, differentiation, and metabolism [61]. In this study, DEGs were significantly enriched in the insulin-signaling pathway in the 4W vs. 16W comparison, suggesting that there were major differences between 4 and 16 weeks of age in the processes of protein synthesis, glucose metabolism, and cell growth. Three enriched cell junction-related pathways (ECM-receptor interaction, focal adhesion, and tight junction) were screened out in the present study, indicating that pathways involved in maintaining the integrity of tissues might be critical for the early growth of chicken. The identification of the first of these pathways can be explained by ECM components playing crucial roles in the formation of the muscle niche. Their specific interactions with muscle satellite cells would affect cell localization, activation, apoptosis, proliferation, and differentiation [62]. In the case of focal adhesions, large, dynamic protein complexes, these are targeted by biochemical and mechanical stimuli from the extracellular environment and can evoke crucial developmental and injury response mechanisms, such as cell growth, movement, and differentiation. Focal adhesions have been considered as mechanical linkages to the ECM [63]. The expression patterns of DEGs belonging to these two pathways (including FN1, PIK3R3, COL6A3, and COL6A1) were very similar, showing the highest expression level at 12 weeks of age, which indicates that there is considerable capacity for satellite cell activities and skeletal muscle development at 12 weeks of age compared with those at other stages. The tight junction is a cellular structure that functions as a barrier to restrict the free passage and movement of ions, liquids, proteins, and larger solutes through the paracellular pathway [64, 65]. This barrier function is essential for the development of multicellular organisms [66]. Another role of the tight junction has been unraveled, namely, that it is involved in the control of cell proliferation and gene expression [67]. In a previous study, it was proposed that tight junction complexes are associated with reorganization of the actin cytoskeleton [68]. The actin cytoskeleton mediates various essential biological functions in all eukaryotic cells [69]. Its dynamic properties play a crucial role in normal cellular processes, including the formation of cellular structures, cytokinesis, adhesion, migration, neurite outgrowth, endocytosis, and phagocytosis [70, 71]. Tight junction and regulation of actin cytoskeleton pathways were both found in the comparison of 12W and 16W, suggesting that these two pathways contribute to the difference in growth rate between 12 and 16 weeks of age.

The expression patterns of DEGs corresponding to these pathways were illustrated by hierarchical clustering analysis (Fig 5). Several gene families or gene subunits were identified among these DEGs, such as the FGF, MYH, and MYL families and subunits of the phosphorylase kinase (PhK) complex. The family of fibroblast growth factors (FGFs), members of which are present in almost all tissues and organs, originally discovered as stimulators of fibroblast or epithelial cell growth, but now fibroblast growth factors are known to exert a broad range of biological activities during vertebrate development. FGFs regulate a number of developmental processes, including brain patterning, branching morphogenesis, and limb development [72]. For example, previous studies indicated that FGF2 was necessary for cell proliferation and neurogenesis in the developing cerebral cortex [73], FGF4 was required for the integration of growth and patterning in the developing limb [74], and FGF12 and FGF13 function in the development of the central and peripheral nervous systems, connective tissue of the skeleton, and the myocardia of the heart [75]. In our study, several members of the FGF family, including FGF2, FGF12, FGF13, and FGF16, were identified with different expression patterns, suggesting their distinct functions and different effects in chicken growth at different stages. Myosin is the most abundant protein expressed in striated muscle cells, consisting of a heavy chain (MYH) and a light chain (MYL) [76, 77]. Multiple members of MYH and MYL gene family have been found differently expressed in this study, including MYH10, MYH1A–F, MYL10, and MYL12A, which have already been reported to be associated with the growth and development of muscle [78, 79]. The diverse protein isoforms of MYH and MYL encoded by specific genes in the families provide the molecular basis of a muscle fiber’s functional diversity [77]. In addition, a previous study reported that different MYH isoforms are expressed in a tissue-specific and developmental stage-specific manner in chicken [80]. MYH1 is critically important for skeletal muscle development, and was found to be closely associated with the protein myosin light chain, phosphorylatable, fast skeletal muscle MYLPF which was inferred to be important as muscle growth occurs mainly in young age in previous study [76, 78]. In this study, MYH1A–F, six isoforms of the MYH1 in chicken, showed similar expression patterns, which were downregulated at 16weeks of age compared to 4 and 12 weeks of age. Thus, it was proposed that these isoforms have similar functions and play critical roles in the growth of leg muscle at the earlier stages. Studies in the past showed that MYH10, a non-muscle myosin, regulated actin cytoskeleton remodeling and was critical for cardiac and brain development [79, 81]. In this study, the opposite expression patterns of MYH10 compared with MYH1A–F indicated its entirely different role in muscle development during the early growth in chicken. PHKA1, PHKB, and PHKG1 are three subunits of the PhK complex, which catalyzes the Ca^2+^-dependent phosphorylation of glycogen phosphorylase in skeletal muscle and stimulates the breakdown of glycogen to ensure a continuous energy supply for cellular activities, including cell migration and proliferation [82, 83]. In a previous study, PhK deficiency was confirmed to induce growth retardation in early childhood [84]. Therefore, the lower expression of these three genes at 16 weeks of age, as identified in this study, may contribute to chicken growth retardation at this stage.

Taken together, the findings in this study have revealed the global changes in the chicken muscle transcriptome during early growth and identified functional categories of genes that contribute to the differences in growth performance among different growth stages. Moreover, the series of potential candidate genes obtained in this study may provide a foundation for further investigation on methods of controlling chicken growth at different stages. Our results should provide an important resource to clarify the mechanisms underlying early growth in chicken.

## Supporting information

S1 Fig Detailed distribution of reads mapped to the reference genome. CDS, coding sequence; UTR, untranslated regions; TSS_up, upstream of the transcription starting site; TES_down, downstream of the transcription ending site.
(TIF)

S2 Fig Directionality of DEGs. 4W vs. 12W, 4W vs. 16W and 12W vs. 16W indicate the comparisons between 4 and 12 weeks of age, between 4 and 12 weeks of age and between 12 and 16 weeks of age, respectively. In each comparisons, up-regulated indicates that the expression in the second group was higher than that in the first group, while down-regulated indicates that the expression in the first group was higher than that in the second group.
(TIF)

S3 Fig Standard curves for primer pairs of the nine genes. (PDF)

S1 Table Primers used for the validation of DEGs by qRT-PCR. (XLSX)

S2 Table The data of chicken growth from 0 to 25 weeks of age. ABW and ADG indicate the average body weight and average daily gain of Jinghai Yellow chicken.
(DOCX)

S3 Table Detailed information on DEGs in the three comparisons of 4W vs. 12W, 4W vs.16W and 12W vs. 16W. (XLSX)

S4 Table DEGs that were uniquely expressed in one of the two groups in each comparisons. The zero besemean value is regarded as no expression in the samples.
(XLSX)

S5 Table Significantly enriched GO terms in DEGs in the three comparisons. (XLSX)

S6 Table Significantly enriched KEGG pathways in DEGs in the three comparisons. (XLSX)
