# Supplementary figures and images for: Transcriptomic profile of leg muscle during early growth in chicken
Source: PLoS One. 2017 Mar 14;12(3):e0173824. doi: 10.1371/journal.pone.0173824 (PMC5349469; doi:10.1371/journal.pone.0173824)

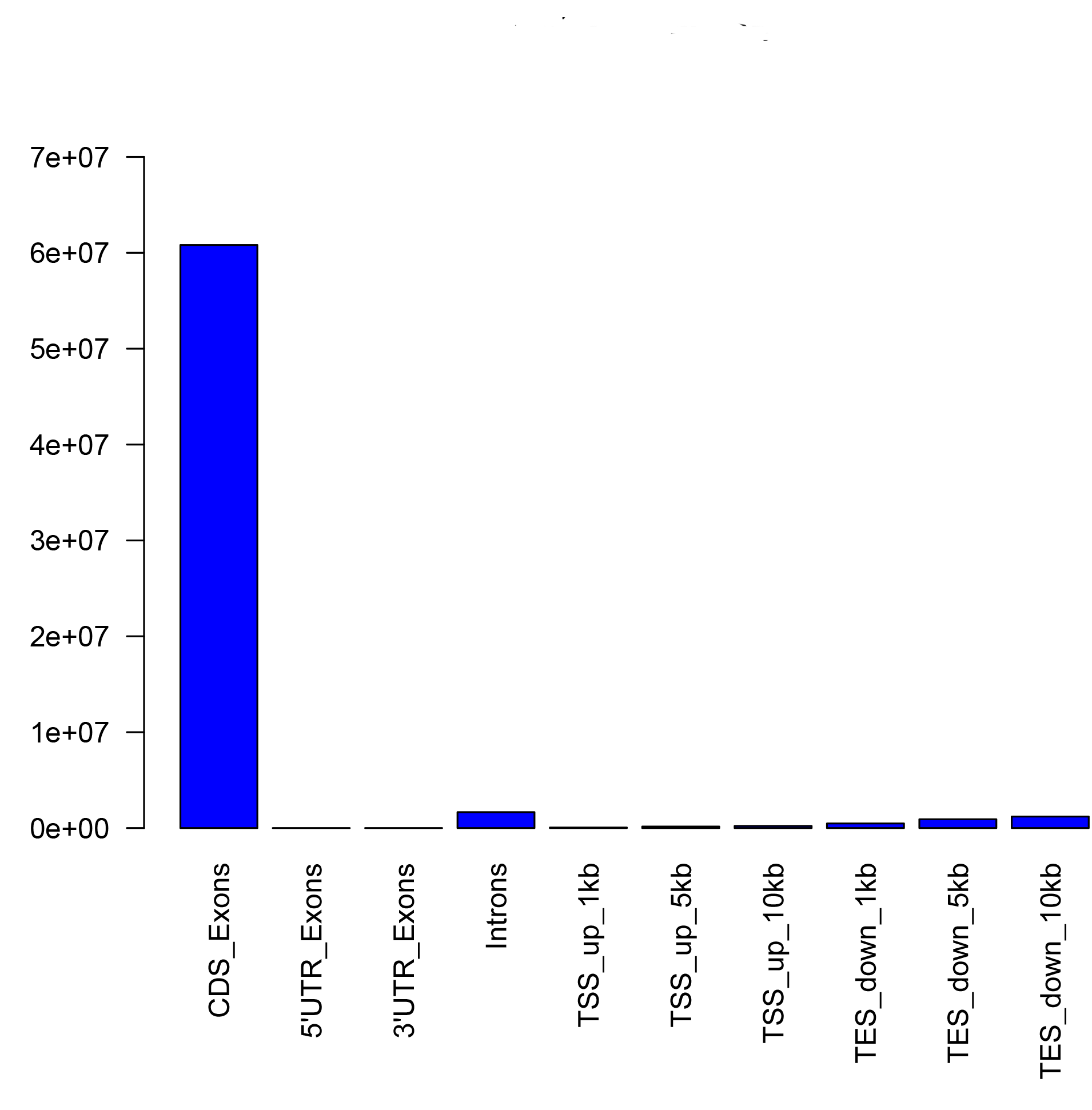

Supplement: S1 Fig — CDS, coding sequence; UTR, untranslated regions; TSS_up, upstream of the transcription starting site; TES_down, downstream of the transcription ending site. (TIF) [file pone.0173824.s001.tif]

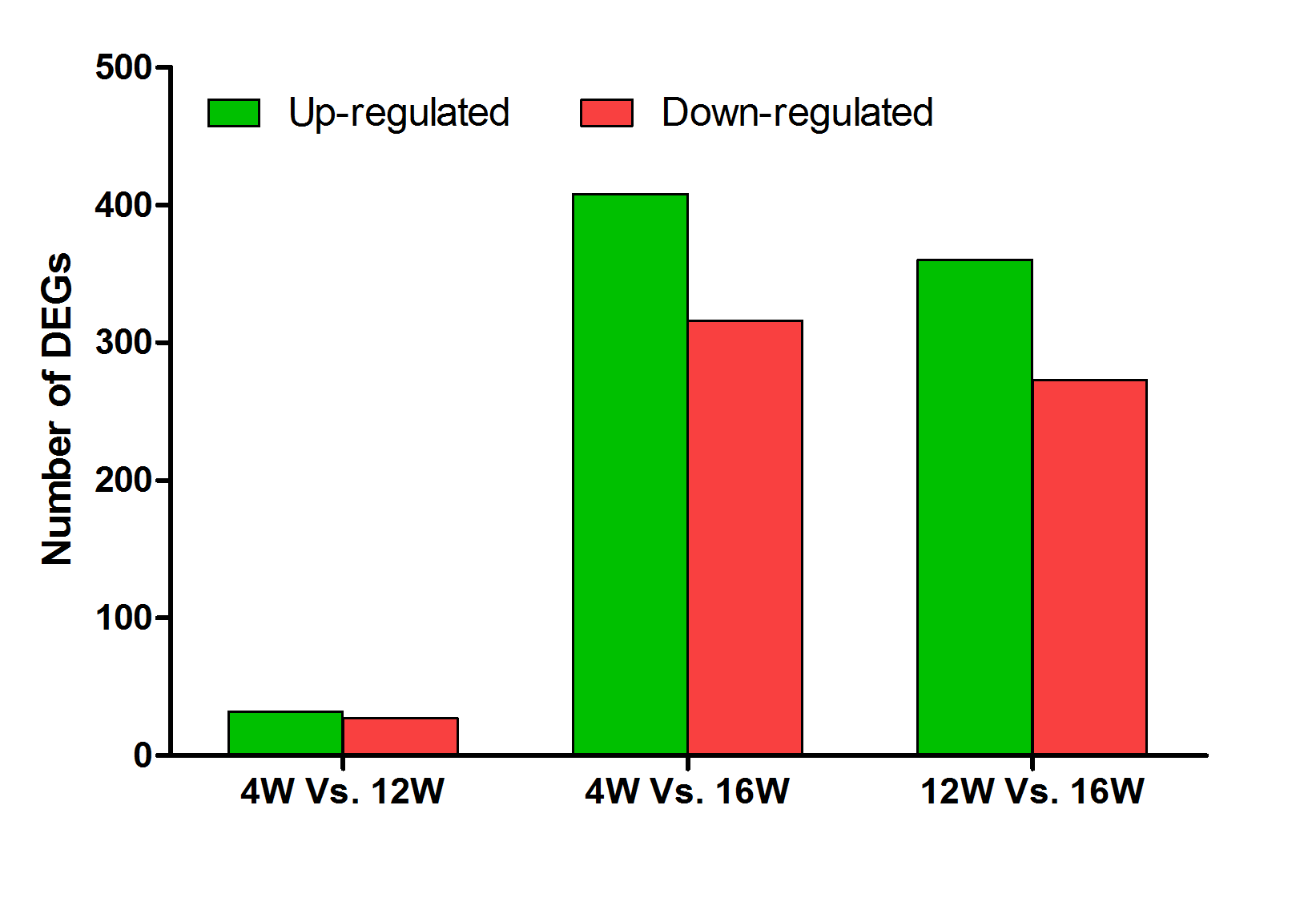

Supplement: S2 Fig — 4W vs. 12W, 4W vs. 16W and 12W vs. 16W indicate the comparisons between 4 and 12 weeks of age, between 4 and 12 weeks of age and between 12 and 16 weeks of age, respectively. In each comparisons, up-regulated indicates that the expression in the second group was higher than that in the first group, while down-regulated indicates that the expression in the first group was higher than that in the second group. (TIF) [file pone.0173824.s002.tif]

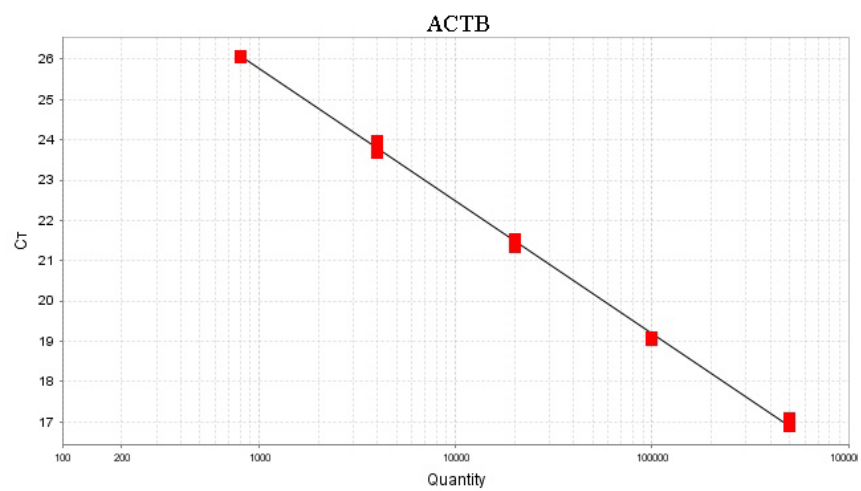

Slope:-3.285 Y-inter:35.61  $R^2$ :0.999 Eff%:101.537

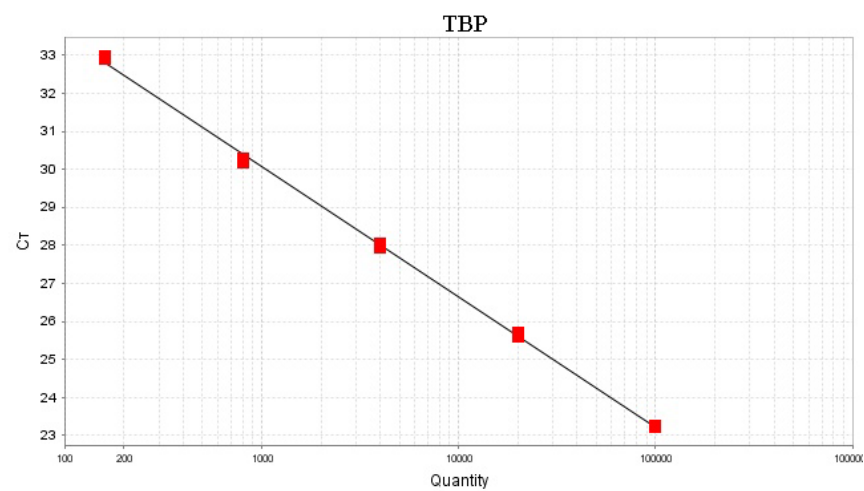

Slope:-3.43 Y-inter:40.364  $R^2$ :0.999 Eff%:95.699

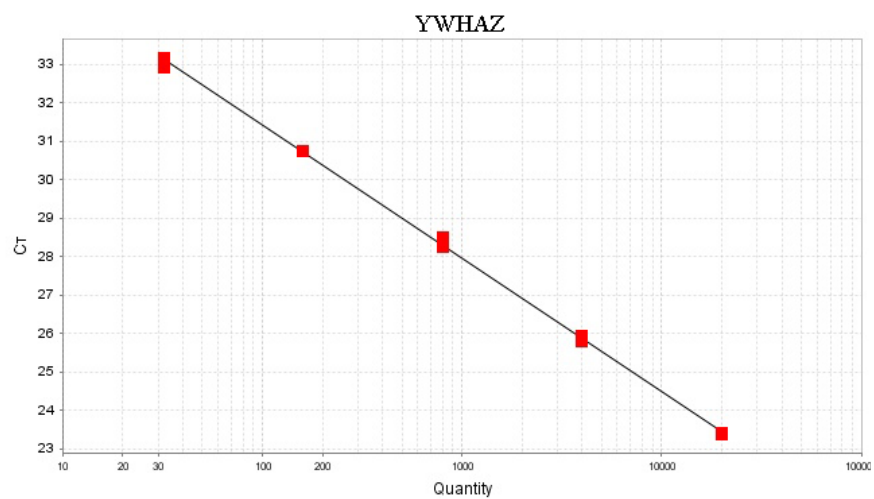

Slope:-3.457 Y-inter:38.312  $R^2$ :0.999 Eff%:94.671

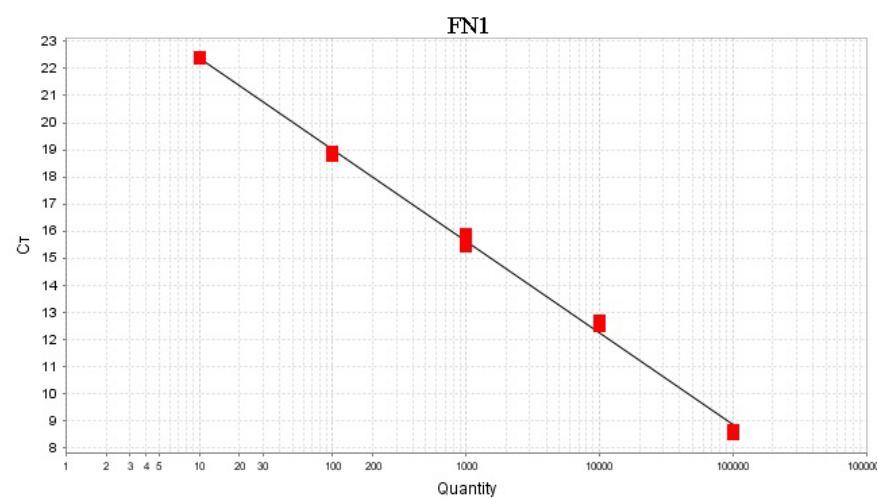

Slope:-3.379 Y-inter:25.741  $R^2$ :0.998 Eff%:97.656

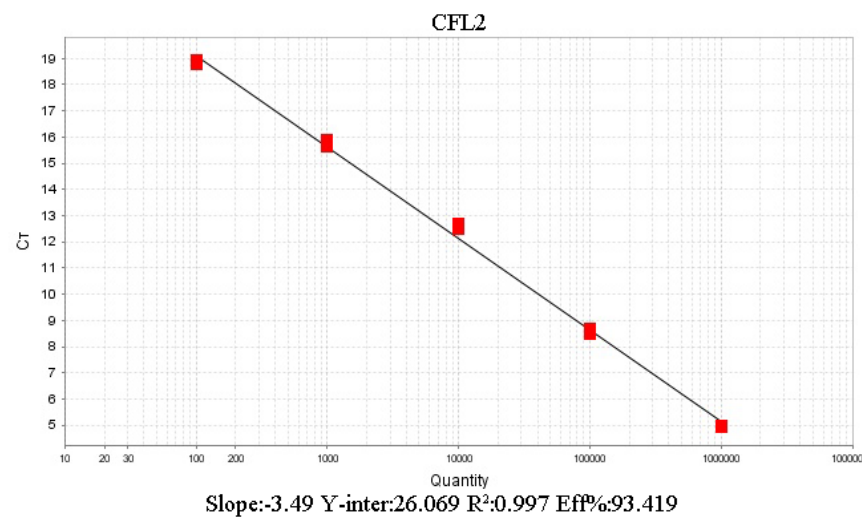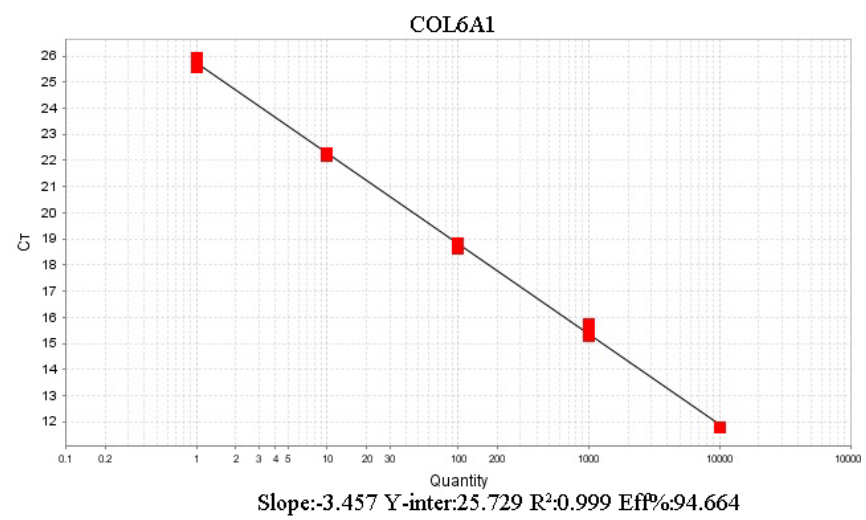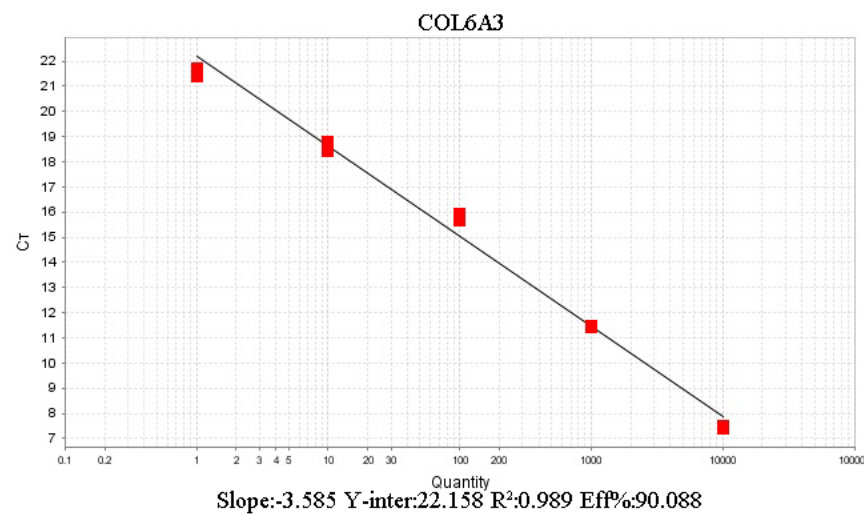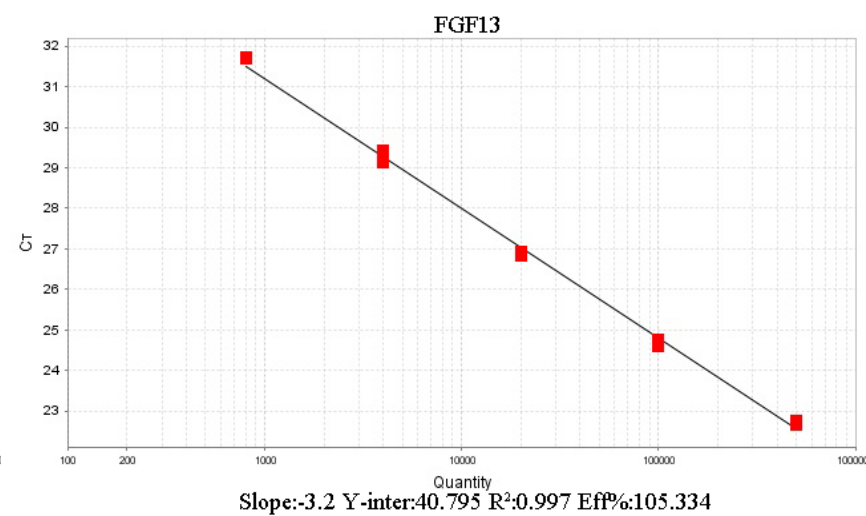

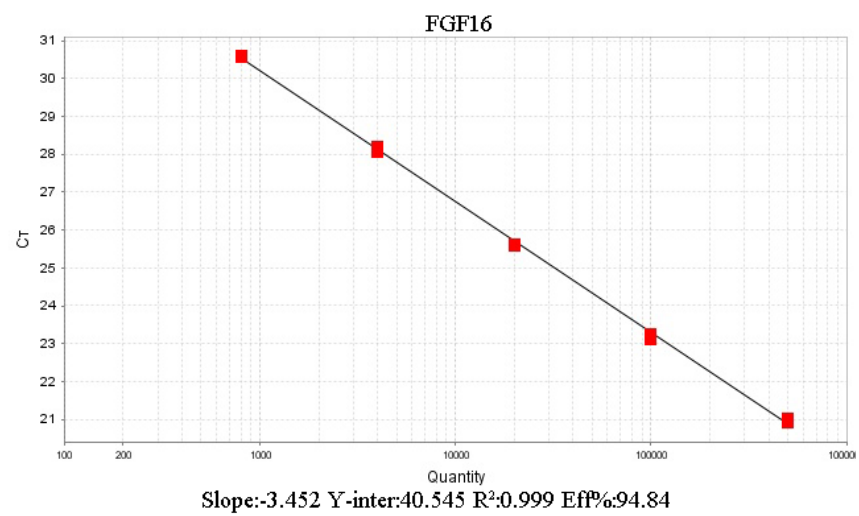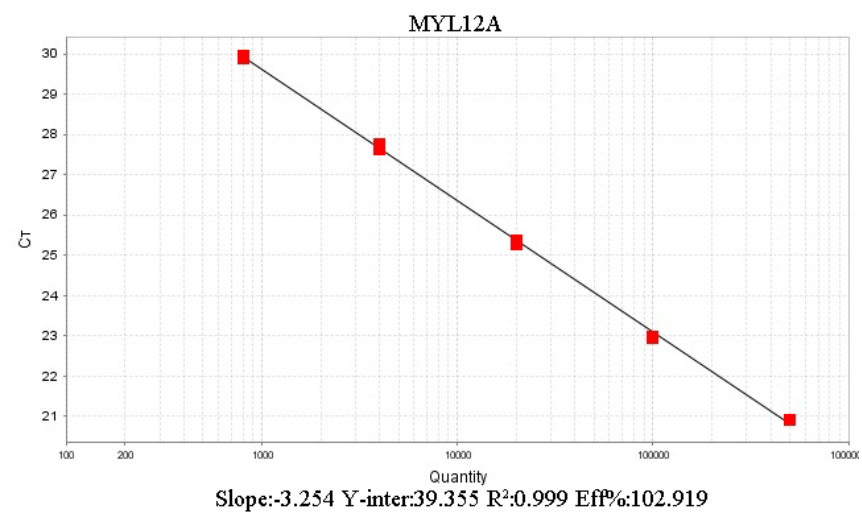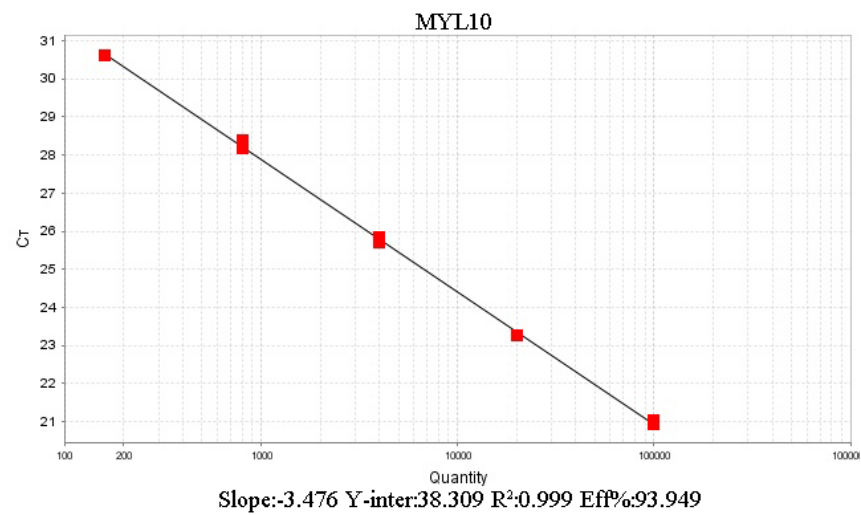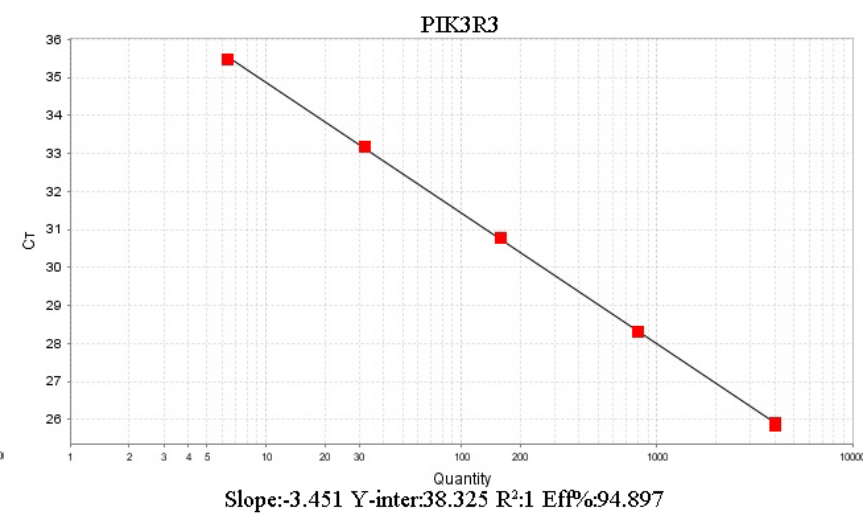

Supplement: S3 Fig — (PDF) [file pone.0173824.s003.pdf]
