# Supplementary material for: Transcriptomic profile of leg muscle during early growth in chicken
Source: PLoS One. 2017 Mar 14;12(3):e0173824. doi: 10.1371/journal.pone.0173824 (PMC5349469; doi:10.1371/journal.pone.0173824)
Supplement: S2 Table — ABW and ADG indicate the average body weight and average daily gain of Jinghai Yellow chicken. (DOCX) [file pone.0173824.s005.docx]

S2 Table . The average body weight (ABW) and average daily gain (ADG) of Jinghai Yellow chicken from 0 to 25 weeks of age

| Weeks of age | ABW (g) | ADG (g) | Weeks of age | BW(g) | ADG (g) |
| --- | --- | --- | --- | --- | --- |
| 0 | 36.45 |  | 13 | 1060.00 | 14.57 |
| 1 | 68.74 | 4.61 | 14 | 1135.00 | 10.71 |
| 2 | 107.00 | 5.47 | 15 | 1198.95 | 9.14 |
| 3 | 148.56 | 5.94 | 16 | 1257.00 | 8.29 |
| 4 | 188.73 | 5.74 | 17 | 1329.00 | 10.29 |
| 5 | 279.98 | 13.04 | 18 | 1375.00 | 6.57 |
| 6 | 350.11 | 10.02 | 19 | 1436.00 | 8.71 |
| 7 | 427.62 | 11.07 | 20 | 1472.00 | 5.14 |
| 8 | 511.46 | 11.98 | 21 | 1511.75 | 5.68 |
| 9 | 604.00 | 13.22 | 22 | 1521.13 | 1.34 |
| 10 | 710.38 | 15.20 | 23 | 1560.3 | 5.60 |
| 11 | 831.00 | 17.23 | 24 | 1575.51 | 2.17 |
| 12 | 958.00 | 18.14 | 25 | 1582.28 | 0.97 |
